# A novel neural response algorithm for protein function prediction

**DOI:** 10.1186/1752-0509-6-S1-S19

**Published:** 2012-07-16

**Authors:** Hari Krishna Yalamanchili, Quan-Wu Xiao, Junwen Wang

**Affiliations:** 1 Department of Biochemistry, LKS Faculty of Medicine, The University of Hong Kong, Hong Kong SAR, China; 2 Shenzhen Institute of Research and Innovation, The University of Hong Kong, Shenzhen, China; 3 Department of Mathematics, City University of Hong Kong, Hong Kong SAR, China; 4 Centre for Genomic Sciences, LKS Faculty of Medicine, The University of Hong Kong, Hong Kong SAR, China

## Abstract

**Background:**

Large amounts of data are being generated by high-throughput genome sequencing methods. But the rate of the experimental functional characterization falls far behind. To fill the gap between the number of sequences and their annotations, fast and accurate automated annotation methods are required. Many methods, such as GOblet, GOFigure, and Gotcha, are designed based on the BLAST search. Unfortunately, the sequence coverage of these methods is low as they cannot detect the remote homologues. Adding to this, the lack of annotation specificity advocates the need to improve automated protein function prediction.

**Results:**

We designed a novel automated protein functional assignment method based on the neural response algorithm, which simulates the neuronal behavior of the visual cortex in the human brain. Firstly, we predict the most similar target protein for a given query protein and thereby assign its GO term to the query sequence. When assessed on test set, our method ranked the actual leaf GO term among the top 5 probable GO terms with accuracy of 86.93%.

**Conclusions:**

The proposed algorithm is the first instance of neural response algorithm being used in the biological domain. The use of HMM profiles along with the secondary structure information to define the neural response gives our method an edge over other available methods on annotation accuracy. Results of the 5-fold cross validation and the comparison with PFP and FFPred servers indicate the prominent performance by our method. The program, the dataset, and help files are available at http://www.jjwanglab.org/NRProF/.

## Background

Recent advances in high-throughput sequencing technologies have enabled the scientific community to sequence a large number of genomes. Currently there are 1,390 complete genomes [1] annotated in the KEGG genome repository and many more are in progress. However, experimental functional characterization of these genes cannot match the data production rate. Adding to this, more than 50% of functional annotations are enigmatic [2]. Even the well studied genomes, such as *E. coli *and *C. elegans*, have 51.17% and 87.92% ambiguous annotations (putative, probable and unknown) respectively [2]. To fill the gap between the number of sequences and their (quality) annotations, we need fast, yet accurate automated functional annotation methods. Such computational annotation methods are also critical in analyzing, interpreting and characterizing large complex data sets from high-throughput experimental methods, such as protein-protein interactions (PPI) [3] and gene expression data by clustering similar genes and proteins.

The definition of biological function itself is enigmatic in biology and highly context dependent [4-6]. This is part of the reason why more than 50% of functional annotations are ambiguous. The functional scope of a protein in an organism differs depending on the aspects under consideration. Proteins can be annotated based on their mode of action, i.e. Enzyme Commission (EC) number [7] (physiological aspect) or their association with a disease (phenotypic aspect). The lack of functional coherence increases the complexity of automated functional annotation. Another major barrier is the use of different vocabulary by different annotations. A function can be described differently in different organisms [8]. This problem can be solved by using ontologies, which serve as universal functional definitions. Enzyme Commission (E.C) [9], MIPS Functional Catalogue (FunCat) [10] and Gene Ontology (GO) [11] are such ontologies. With GO being the most recently and widely used, many automated annotation methods use GO for functional annotation.

Protein function assignment methods can be divided into two main categories - structure-based methods and sequence-based methods. A protein's function is highly related to its structure. Protein structure tends to be more conserved than the amino acid sequence in the course of evolution [12,13]. Thus a variety of structure-based function prediction methods [14,15] rely on structure similarities. These methods start with a predicted structure of the query protein and search for similar structural motifs in various structural classification databases such as CATH [16] and SCOP [17] for function prediction. Structural alignments can reveal the remote homology for 80-90% of the entries in Protein Data Bank [18] even if no significant sequence similarity was found for the two proteins [19]. However, these methods are limited by the accuracy of the initial query structure prediction and the availability of the homologous structures in the structural databases. Despite of being highly accurate, the big gap between the number of sequences and their solved structures restricts the use of structure-based methods. Therefore, sequence-based methods are needed.

The main idea behind sequence-based methods is to compare the query protein to the proteins that are well characterized, and the function of the best hit is directly assigned to the query sequence. GO annotations are assigned to the BLAST search results [20] for the first time by GOblet [21] which maps the sequence hits to their GO terms. Later on the GO terms are given weights based on the *E-value *of the BLAST search by Ontoblast [22]. This was further refined in GOfigure [23] and GOtcha [24] by communicating the scores from one level to the other in the GO hierarchy tree. All these methods are based on the BLAST search results; thus they fail to identify the remote homologues with a higher *E-value*. This problem is tackled by the Protein Function Prediction (PFP) server [25], which replaces the BLAST with PSI-BLAST [26] and thus can detect remote homologues. The PFP server can predict the generalized function of protein sequences with remote homology, but with a trade-off of low specificity. FFPred [27] is the most recent protein function prediction server that builds Support Vector Machine (SVM) classifiers based on the extracted sequence features of the query sequence and thus it does not require prior identification of protein sequence homologues. However the server needs one SVM classifier for each GO term, which makes it computationally expensive. Furthermore, the server only provides classifiers for 111 Molecular function and 86 Biological Process categories that represent more general annotations, which limits its usage in deciphering specific annotations. The lack of annotation specificity and high complexity of the existing methods advocate the need of improvement in the automated protein function prediction.

**Figure 1:**
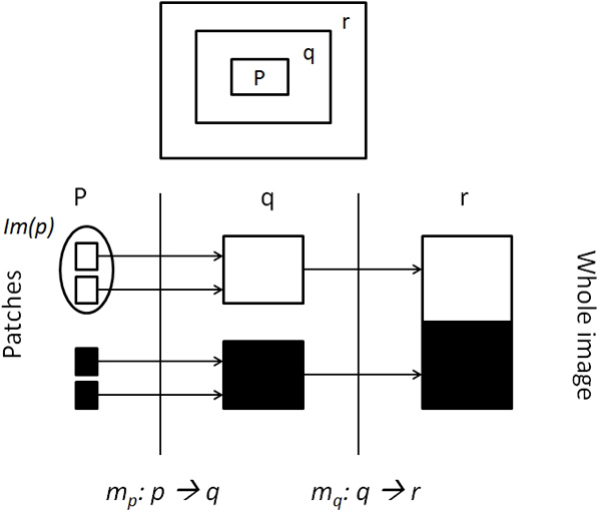
**Three layer mode for image classification**.

Here we present a novel automated protein functional assignment method based on the neural response algorithm [28]. The algorithm simulates the neuronal behavior of human's image recognition, and has been successfully applied for image classification. The main idea of this algorithm is to define a distance metric that corresponds to the similarity of small patches of the images and reflects how the human brain can distinguish different images. This algorithm uses a multi-layer framework with spatial scale, and size increasing as we move from the one layer to the other in a bottom-up fashion. The bottom layer consists of templates (sub-patches) of the images and the intermediate layers consist of secondary templates formed by the assembly of the templates in the lower layers. The whole image is in the topmost layer. For example consider a three layered architecture of templates (patches) *p*, *q *and *r *(whole image), with *p *⊂ *q *⊂ *r *as shown in Figure 1. Let *Im(p)*, *Im(q) *and *Im(r) *be the function spaces corresponding to the similarity of the templates in the layers *p*, *q *and *r *respectively. *Im(x) *gives the similarity between any two patches in the layer *x *and a mapping set *m*: that maps the templates from the bottom most layer to the templates in the next layer i.e. *m_p_: p → q*, and similarly *m_q_: q → r*. Having defined the layers (*p, q and r*) and the initial layers similarity function *Im(p)*, the algorithm builds a derived kernel on the top of layer *r *in a bottom-up fashion. The process starts with the calculation of initial reproducing kernel *k_p _*on the bottom most layer *p *as the inner product of its functional space *Im(p)×Im(p)*. Based on the this initial kernel *k_p_*, intermediate derived kernel *k_q _*is computed on top of the layer *q *and this in turn is used to compute the final derived kernel *k_r _*on the top most layer *r*, which can help us in the classification of the whole images in layer *r*. Refer to [28], for the detailed mathematical formulation of the initial and the derived kernels. The computation of kernels forms the unsupervised preprocessing component and is key for the superior performance of the neural response algorithm as it can minimize the complexity of the corresponding image classification problem (supervised task)[28].

In the current context of protein functional characterization, the top layer represents the whole protein sequences and the subsequent layers are constituted of sequence motifs. At each layer similarity is computed between the templates of two successive layers, which are referred to as derived kernels by taking the maximum of the previously computed local kernels in a recursive fashion. Finally a mapping engine is built on the kernels derived from the neural response algorithm to map the query protein to its most probable GO term. A detailed description of the whole methodology is given in the Methods section.

## Results

We used the GO terms with no further children (leaf nodes of the GO tree) and their corresponding proteins for the assessment of our method. The rationale for using leaf nodes is that these GO terms are functionally more specific than the GO terms at the higher levels, i.e. no two GO terms should share a common protein and thus can demonstrate the specific function prediction strength of our method. This also addresses the issue of redundancy in the training set. To further fortify our argument we had also addressed the redundancy problem at sequence level by eliminating the redundant sequences that are more than 80% similar in the training set. This was done by using CD-HIT [29], a program that removes redundant sequences and generates a database of only the representatives. From the extracted GO terms we enumerated all the protein pairs belonging to the same GO term and labeled them as positive dataset i.e. we assigned a label Y*_(i, j) _*as 1 and the protein pairs belonging to different GO terms were labeled as negative, Y*_(i, j) _*= 0. Among such labeled pairs, we randomly selected 3000 positive pairs and 3000 negative pairs and used these labeled protein pairs to train and validate our method. After training the final mapping function, *f(N_(i, j)_) *produced a value between 0 and 1 corresponding to the similarity between the proteins *i *and *j *in the validation set. Upon applying the threshold of 0.5, we predicted the labels Y*_(i, j) _*to 1 (share a GO term) if *f(N_(i, j)_) ≥ 0.5*, and predict Y*_(i, j) _*to 0 (do not share a GO term) if *f(N_(i, j)_) < 0.5*.

### Cross validation

To evaluate our method we performed 5-fold cross validation i.e. we randomly divided the pool of 6000 labeled protein pairs into five partitions with an equal number of positive and negative labeled pairs. Out of the five partitions, four were used to train the neural response algorithm, and the remaining one partition was used to test the algorithm. This process was repeated for five time (the *folds*), with each of the five partitions used exactly once as the validation data. The idea was to check whether our method can correctly classify the pairs, which were not used for training. The values of average accuracy, area under the curve (AUC) and training time of the 5-fold cross validation are reported in Table 1, with respect to the template library and the mapping engine used (See Methods). The difference in the accuracies using the PROSITE and PFAM template libraries is due to the differences in the respective sequence coverage. Thus we combined the PFAM and PROSITE templates for a better sequence coverage, and indeed, the accuracy increased (Table 1). Out of the two mapping engines Least Squares classifier is almost 3 folds faster than the SVM classifier with almost the same accuracy (Table 1). Therefore we report the accuracy values using the Least Squares mapping engine.

**Table 1:** 5 Fold cross validation results with respect to the template library

|S|	Template Library in layer 2	SVM		LS	
		
		Accuracy	AUC	Accuracy	AUC
1	PROSITE	77.1%	0.851	76.4%	0.863
2	PFAM	80.5%	0.875	80.2%	0.881
3	PROSITE + PFAM	82.0%	0.882	81.70%	0.892
	Training Time	151.9 Sec.*		54.9 Sec.*	

### Classification specificity with respect to the GO term distance

**Figure 2:**
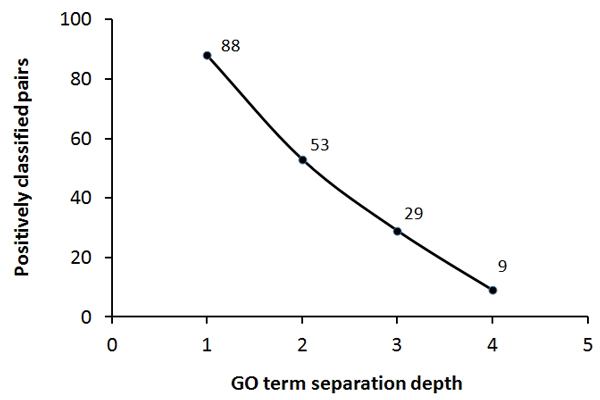
**Classification specificity plot**.

As described in methods, the derived kernel classifies two proteins to be similar, if the pair is equivalent (similar) to a pair with two known similar proteins. To test the classification specificity of our method, we have selected 800 proteins (400 pairs) with the first 100 pairs sharing an immediate parent GO term (level 1); second 100 pairs sharing a common parent separated by an edge distance of 2 in the GO tree (level 2). Similarly we have level 3 and 4 datasets with an edge distance of 3 and 4 respectively. As the positive pairs in the training set share a common GO term, we expect our method to classify the protein pairs as positive whose GO terms are the same or the next one in the GO hierarchy and as negative if their respective GO terms are far away. The number of positively classified (similar) pairs in respective subsets is given the Figure 2. We observed that the proportion of positively classified (similar) pairs is 88% in the level 1 dataset as they are much closer in the GO tree and it gradually dropped to 9% in the level 4 dataset as the GO distance between them is increased. This suggests that our method is highly specific in classifying the similar proteins with respect to the relative distance between the respective GO terms.

### Comparison of classification accuracy

Having shown the predominant classification specificity and the 5 fold cross validation results, we further compare the classification accuracy of our method with the PFP and FFPred servers, which are the most sensitive protein function prediction server using GO vocabulary [3] to date. We had compiled a test set of 400 proteins constituting of 200 protein pairs, with 100 pairs sharing the same GO term (positive test set) i.e. the edge distance between the GO terms of a protein pair is zero and other 100 pairs sharing a distant root GO term (negative test set) i.e. the edge distance between the GO terms of a protein pair is ≥ 1. Each of the 200 protein pairs were classified as either positive (similar) or negative (non similar) by NRProF. Since PFP or FFPred server does not have a standalone software version, we had to submit our query directly to the online server manually for each of the 400 proteins. The PFP and FFPred servers list the probable GO terms for a query protein sequence with a confidence score associated with each of the GO terms. A classification is considered to be accurate if the servers predict the same GO term (rank 1) for both the proteins of a pair in the positive test set and different for the negative test set. On the other hand NRProF classification is considered to be accurate if it can classify the positive set as similar and negative set as dissimilar pairs. Out of 200 predictions, NRProF performed better than PFP and FFPred servers in 8 and 5 instances respectively. The accuracies are tabulated in Table 2. We therefore conclude that NRProF has a better classification accuracy.

**Table 2:** Classification Accuracy of the NRProF, FFPred and PFP server with respect to the compiled test set.

|S|	Method	Accuracy
1	NRProF	83.8%
2	FFPred	81.5%
3	PFP Server	80.5%

### GO term predictability

**Figure 3:**
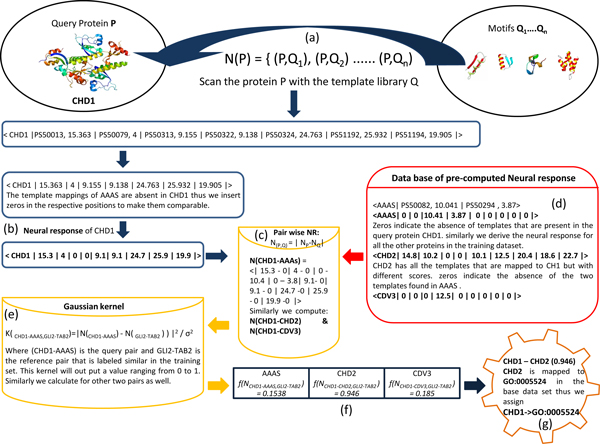
**Detailed workout of CHD1**. (a) Query sequence CHD1 is scanned for the potential template hits. (b) Computation of the corresponding neural response. (c) Calculation of pair wise neural response *N(p, q_j_) viz*. (CHD1-AAAS), (CHD1-CHD2) and (CHD1-CDV3). (d) Database of pre-computed neural response for the base dataset. (e) The pair wise pair wise neural response vectors are fed to the mapping function using a Gaussian kernel to generate a value ranging from 0 to 1. (f) Proteins in the base dataset ate sorted in descending order based on their similarity (*f(N_(i, j)_)*) to the query protein. (g) GO term *GO:0005524 *of CHD2, with high mapping score is assigned to CHD1.

Next we demonstrate the GO term predictability of our method. Our method labels a protein pair *p_i _*(query protein) and *p_j _*(protein in the base dataset) as 1 if they are similar and thereby assigns the GO term of the protein *p_j _*to the protein *p_i _*based on the threshold applied on the function *f(N_(i, j)_)*. To overcome the threshold dependency and to make the results comparable with the PFP and FFPred servers, we had sorted the proteins in the base dataset in descending order based on their similarity (*f(N_(i, j)_)*) to the query protein, and assigned the GO term of the corresponding most similar (rank 1) protein to the query protein. For a better understanding of the methods, we present the stepwise workout of the algorithm for a human protein Chromodomain Helicase DNA binding protein 1 (Figure 3). Firstly the query sequence CHD1 was scanned for the potential template hits. We got 7 hits in the template library, with no hit occurring more than once thus a neural response vector can be computed with equation 4 (see Methods). The neural response vector computed was < CHD1 |PS50013, 15.363 | PS50079, 4 | PS50313, 9.155 | PS50322, 9.138 | PS50324, 24.763 | PS51192, 25.932 | PS51194, 19.905 | >. The first element in the vector is the query protein followed by the template ID (Prosite/Pfam) and its score respectively. However if the query sequence have repeats or if a template t has more than one hit in the query sequence we consider the hit with the maximum score (equation 3, Methods). We then calculated the pair wise neural response *N(p, q_j_) *(equation 5, Methods) where *p *is the query (CHD1) and *q_i _*is the pre-computed neural response of the *i^th ^*protein sequence from the initial base set. For illustration here we show the calculation of 3 pair wise neural response vectors (CHD1-AAAS), (CHD1-CHD2) and (CHD1-CDV3) in the Figure 3. Next these pair wise neural response vectors together with the another pair wise neural response vector (which is known to be similar) were fed to the mapping function using a Gaussian kernel (equation 7, Methods) to generate a value ranging from 0 to 1 corresponding to the similarity between the proteins in the pair wise neural response. Then we sorted the proteins in the base dataset in a descending order based on their similarity (*f(N_(i, j)_)*) to the query protein. Since the *f(N(_CHD1-CHD2_) *is higher than the other two, we assigned the GO term of CHD2 to CHD1. In the GO tree, CHD2 has 22 associated GO terms. Since we considered only leaf GO terms for the higher annotation specificity, we assigned the GO term GO:0005524 (leaf GO term associated with CHD2) to CHD1. However, in addition to the current state of the algorithm if users wish to consider other non-leaf GO terms, we suggest the users to use the sequence diversity (simply the number of representative sequences after the CD-HIT filtration) in the GO terms associated with the most similar protein to the query protein, as a criteria for assigning the GO term i.e., the GO term with the least sequence diversity is assigned to the query protein. This can be advocated by the fact that the sequence diversity is inversely proportional to the specificity of the GO terms. In the current example by either ways our method had assigned the term GO:0005524 to the CHD1.

### Comparison with the existing methods

We compared the GO term predictions of our method with PFP and FFPred servers, which are the most sensitive function prediction servers to date. PFP and FFPred servers predict the most probable GO terms for a query protein with a confidence score associated with each of the GO terms. A prediction is considered to be accurate if actual (most specific) GO term of the query protein is ranked among the top 5 probable GO terms by the respective methods. Lack of standalone versions of PFP and FFPred is a serious limitation on the dataset used for comparison. We compiled a dataset of 300 proteins each belonging to the leaf nodes of the GO tree. The prediction results from PFP and FFpred were obtained by manual submissions to the respective servers. Table 3 compares the GO terms predicted for the Human protein WDR55. PFP could not report the actual leaf GO term in its top 5 predictions. This is due to trade-off of annotation specificity to weak hits with High *e *value. FFPred could not predict any GO term because it is limited to only 111 Molecular function and 86 Biological Process categories. Whereas NRProF predicted top 3 similar proteins with the same GO term. The Overall accuracy on the set of 300 proteins is reported in the Table 4.

**Table 3:** GO terms predicted for the protein Q9H6Y2 by PFP, FFPred and NRProF.

Protein Name/ID	WDR55/Q9H6Y2
**Actual Leaf** **GO term**	GO:0002039

**Top 5 GO terms by PFP**	GO:0005488, GO:0043169, GO:0003676, GO:0004977, GO:0046026

**Top 5 GO terms by FFPred**	No GO terms predicted for this sequence

**Top 5 GO terms by NRProF**	P51532, Q96S44, Q9HCK8 (GO:0002039), Q01638 (GO:0002114), Q13822 (GO:0047391)

**Table 4:** GO term prediction Accuracy of the NRProF and PFP server with respect to the test set.

|S|	Method	Accuracy	AUC
1	NRProF	86.93%	0.9453
2	PFP Server	83.33%	0.8892

From Table 4, we can infer that our method NRProF performs reasonably better than the PFP server. We have not reported the accuracy of the FFPred, as it is limited to only 111 Molecular function categories, which makes it suitable for general rather than specific function annotations. There are other methods that use GO vocabulary for protein function prediction methods including GOblet, GOfigure and GOtcha. But the PFP server has already been proved to be superior to all the above mentioned methods [25]. Thus we have compared our method (NRProF) only with the PFP server.

## Discussion

### Mapping function threshold

**Figure 4:**
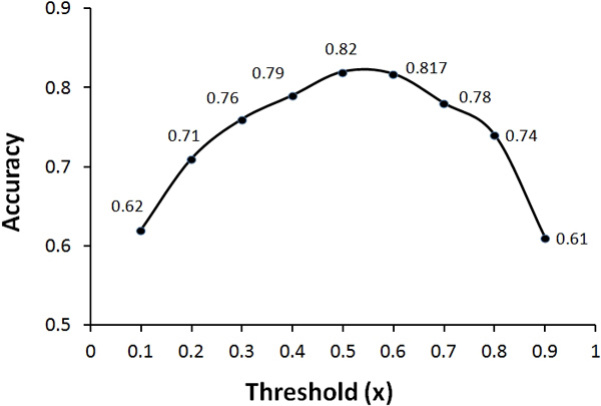
**Accuracy plot for different threshold values**.

The mapping function, *f(N_(i, j)_) *produces a value between 0 and 1 corresponding to the similarity between the proteins *i *and *j*. Upon applying the threshold of 0.5, we assign the labels Y*_(i, j) _*to 1 (share a GO term) if *f(N_(i, j)_) ≥ x*, and to 0 (do not share a GO term) if *f(N_(i, j)_) < x*. We tried different values of *x *to decide on the best threshold. Different threshold values and their corresponding accuracies are plotted in Figure 4. It can be observed that the accuracy is high for the threshold values ranging from *0.5 *to *0.6*. Thus we selected *0.5 *as the optimal cut-off.

### CD-HIT threshold

Here all our validation and test data sets constitute of Human GO terms (same species) thus we need to take care of the redundancy. This was implicitly addressed by using GO nodes with no children; however we even address this issue at the protein sequence level by using CD-HIT with optimal cut-off, to ensure proper training. An optimal threshold should not be too high or too low, if so the predictions will be biased towards highly similar/dissimilar proteins respectively. In order to observe the influence of this cut-off on the accuracy, we compared the accuracy values on a test set of 2000 protein pairs, with 1000 positive and 1000 negative pairs with respect to five different cut-offs and the results are shown in the Table 5. We can observe that the accuracies at 60% and 100% cut-offs are less when compared to others. This may be due to the biased training on negative and positive protein pairs respectively. The accuracies at 70% and 80% are almost as good as or higher than the other cut-offs. This supports the use of 80% as the cut-off to eliminate the redundancy. However this cut-off should be changed with the addition of sequences from the other species. Thus we advise to choose the cut-off based on the diversity of the dataset.

**Table 5:** Impact of CD-Hit cut-off on the accuracy

|S|	CD-Hit cut-off	Accuracy
1	60%	78.3%
2	70%	83.6%
3	80%	84.2%
4	90%	81.7%
5	100%	80.4%

### Similarity based on protein pairs

We can simply calculate the similarity between a query protein and a known one to assign the corresponding GO term. However with this similarity, we can only use some naive algorithms like k-nearest neighborhood, whose accuracy is not quite satisfactory especially for biological data (proteins), which is essentially multi dimensional. In addition to this, we should artificially enforce a similarity cut-off between the query and the known protein to assign the query protein to its associated GO category. Considering the fact that the intra GO term similarity varies from GO term to GO term it is difficult to set such cut-offs. To conquer this, it is necessary to design a machine learning algorithm that can learn and chose the cut-off based on the similarity between the proteins sharing the same GO term i.e. the similarity cut-off should be high if the intra GO term similarity is high and vice versa. Here, our model assigns the query protein to its associated GO category (1^st ^pair) based on the respective Intra GO term similarity, given by the similarity between the proteins constituting the 2^nd ^pair, i.e. the 1^st ^pair will be labeled as similar if its similarity is equivalent to the similarity of the 2^nd ^pair (labeled as similar) and vice versa. By this we can bypass the cut-off that needs to be enforced on the simple similarity score for assigning GO terms.

### GO term mapping

**Figure 5:**
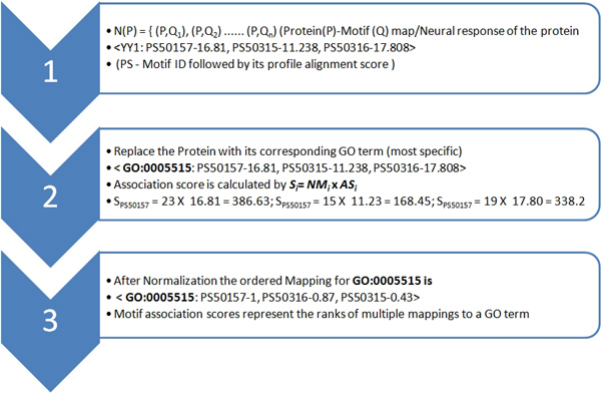
**GO tem mapping with respect to the template library**.

Mapping contains entities from external database system indexed to similar or related GO terms. Currently these mappings in the Gene Ontology database are made manually consuming a lot of resources and time. As a spin-off, our methodology can automate the process of mapping between the templates (Prosite/Pfam) and the GO terms, without compromising much on the accuracy. The neural response of a protein with respect to all the templates computed according to the equations 4 (Methods) is nothing but the mapping of a protein (GO term) to the respective templates (motifs). GO-Motif association scores for the same is given by:

(1) $$s_{i}={\left(NM\right)}_{i}\times {\left(AS\right)}_{i}$$

where *NM_i _*is the number of proteins (after removing the redundancy by CD-HIT) having a specific motif *i *associated to a GO term (motif frequency) and *AS_i _*is the alignment strength of the respective motif's. We use the product of *NM_i _*and *AS_i _*to achieve a trade-off between the overrepresentation of a motif to its alignment strength. The detailed calculation is shown in Figure 5. The computed GO-Motif association scores can be used to rank the multiple mappings to a GO term.

### Proteins with multiple leaf GO terms

Our test set is compiled of leaf GO terms and their corresponding proteins with no two GO terms sharing a common protein, to demonstrate the specific function prediction strength of our method. However, up on perusal we found that ~25% of the proteins belong to more than one leaf GO term under the category of molecular function. To analyse the effect of "not including such proteins" on the accuracy, we have compiled a new test set of the same size (300 proteins including proteins belonging to more than one leaf GO term). We perceive that considering proteins belonging to more than one leaf GO term has no negative effect on the GO term predictability. In fact the prediction accuracy is slightly better 89.63% when compared to 86.93% on the actual test set.

## Conclusions

Here we present a novel protein function prediction method, *NRProF*, based on the neural response algorithm using the Gene Ontology vocabulary. The neural response algorithm simulates the neuronal behavior of the visual cortex in the human brain. It defines a distance metric corresponding to the similarity by reflecting how the human brain can distinguish different sequences. It adopts a multi-layer structure, in which each layer can use one or multiple types of sequence/structure patterns.

*NRProF *is the first instance of neural response being used in the biological domain. It finds the most similar protein to the query protein based on the neural response *N *between the query and the target sequences; and thereby assigns the GO term(s) of the most similar protein to the query protein. This is a profound and composite method with the essence of sequential, structural and evolutionary based methods for protein function prediction. The templates from the PRINTS and PFAM database contribute to the functional profiles or signatures (sequence). The mismatch and deletion states in the HMM profiles of the PFAM templates account to the degeneracy due to evolution and the secondary structural information of the match states in the HHM profiles contribute to the structural part. The use of HMM profiles along with the secondary structure information of PROSITE and PFAM sequence motifs to define the neural response gives our method an edge over other available methods to identify the remote homologues, as profile-profile alignments are superior to PSI-BLAST based methods in detecting the remote homologues. Thus NRProF can complement most of the existing methods.

Our method is computationally less complex compared with the other methods, as the initial neural response of the proteins in the base dataset with respect to the template library are computed only once and from there the neural response between the query and target is computed with the least computational effort unlike other BLAST/PSI-BLAST based methods. The simple derived kernel adds to the computational simplicity of our method. We validated our method in a 5-fold cross validation fashion and obtained an accuracy of 82%. Considering the criterion that a prediction is valid if and only if the actual GO term is top ranked (1^st ^Rank) GO term by our method, 82% is quite a good accuracy. The classification accuracy of 83.8% on a test set of 400 proteins suggests that our method is highly specific in classifying the similar proteins with respect to the relative distance between the respective GO terms. Upon further caparison of our method with the PFP and FFPred servers which are the most sensitive function prediction servers to date, the GO term prediction accuracy of 86.93% evince that our method is more accurate in predicting the specific functions. Thus we conclude that our method is computationally simple yet accurate when compared with the other methods. This is achieved by simulating the neuronal behavior of the visual cortex in the human brain in the form of neural response.

## Methods

**Figure 6:**
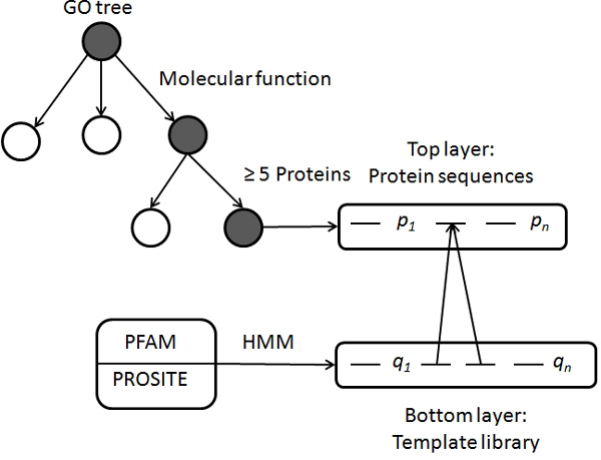
**Two layers of the model and their respective data sources**.

The neural response algorithm can be viewed as a multi-layered framework as described in the background section. Here we built a two layer model as shown in Figure 6, with the whole protein sequences in the top most layer and the templates (sequence motifs) in the subsequent layer. We used Gene Ontology (GO) vocabulary for protein functional assignment, i.e. we mapped the query protein to its corresponding GO term(s) that represent(s) the properties of the query sequence. GO terms covers three major domains: cellular component, molecular function, and biological process. We downloaded the ontology file (OBO) v1.2 from the GO resource.

To demonstrate our approach, we only used the molecular function domain with a total of 8,912 GO terms. Then we extracted the proteins and their sequences belonging to each of the GO terms. To address the issues of redundancy we had used CD-HIT [29], a program that removes redundant sequences and generate a database of only the representatives. These protein sequences and their respective GO terms were used as the base dataset for our model. We only used proteins from humans because we wanted to demonstrate the ability of our method to predict/characterize the function of the proteins even if they are remotely homologous to the pre-characterized proteins (human).

**Figure 7:**
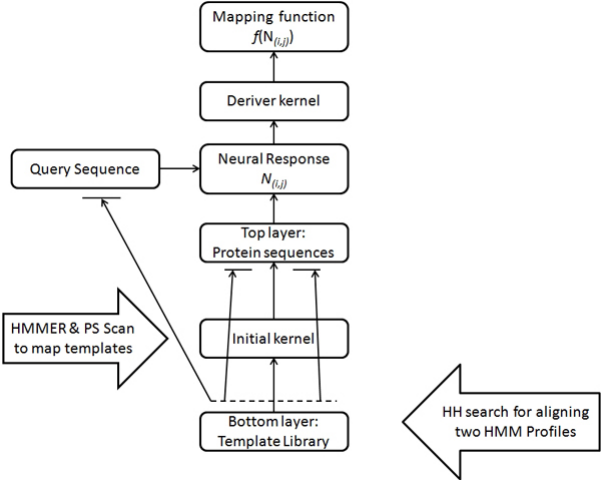
**Pipeline diagram showing the control flow of the method**.

We further trimmed our GO terms by screening out the terms with less than 5 proteins. The resultant GO terms form the base set for our method and their associated proteins form the top layer in the model. For the bottom layer (template library), we used the sequence motifs from PROSITE [30] version-20.68 and Pfam [31] version-24. The rationale behind choosing PROSITE and Pfam is that Pfam has the largest sequence coverage [3] and PROSIRE has small sequence motifs that can be useful in detecting remote homologues in the absence of a whole conserved domain. We downloaded the PROSITE patterns and Pfam domains as Hidden Markov Model (HMM) [32] files from the respective repositories. Here we built two kernels, one on the top of each layer. First an *initial kernel *is computed on top of the template layer, which can be used as a similarity function between the templates. Then a *derived kernel *is computed on top of the top layer by choosing the maximum neural response between the individual templates in bottom layer and the sequences in top layer. Computation of the initial kernel, the neural response and the derived kernel is explained in detail in the following subsections and the overall pipeline of the methodology is shown in Figure 7.

### Initial kernel

Let there be m templates (sequence motifs) *q_1_...q_m _*in the bottom layer. We need to define a non-negative similarity measure *s(q_i_, q_j_) *between any two motifs *q_i _*and *q_j_*. A natural condition for similarity is *s(q_i _, q_j_) ≤ s(q_i _, q_i_) *for any *q_i _≠ q_j_*, which means a motif is always more similar to itself than to the others. Besides this, to ensure the validity of our algorithm, a mathematical requirement of the similarity is that for a set of motifs *q_1_...q_m_*, the matrix *S *should be a positive definite matrix.

(2) $$S={\left[s\left(q_{i},q_{j}\right)\right]}_{i,j=1}^{n}$$

Our template library in bottom layer consists of HMM profiles from the Pfam database, thus we define the similarity between templates as profile-profile alignment scores. We had 10,257 profiles in the template library, making ~10^6 ^profile-profile alignments. To align the template HMM profiles we used HHsearch which is the most sensitive profile-profile alignment tool to date [33-35]. As a refinement for better sensitivity and to capture the remote homology between the templates, we considered the secondary structure information of the templates as well, which is considered more conserved and provides additional information [36]. We have previously used secondary structure information to improve protein sequence alignment [37] and remote homologue identification [38]. Thus we converted the HMM profiles to HHM [34] profiles containing the secondary structure information of all the match states in the HMM profiles. We employed HHsearch which uses PSI-PRED [39] to predict the secondary structure and added them to the HMM profiles. By doing this we were able to capture the remote homologues templates. Profile-Profile alignments were proved to be more sensitive than PSI-BLAST in the identification of remote similarity [40]. Thus our method has the edge over the PFP server which is based on PSI-BLAST in detecting the remote homologues.

### Neural response

Consider a protein *p *in top layer with *k *template hits denoted by *q_p1_...q_pk _*in bottom layer. PrositeScan [41] and HMMER 3.0 [32] are used to scan the protein sequences in top layer with the templates from PROSITE and Pfam respectively. Both PrositeScan and HMMER 3.0 were used in the local alignment mode as here we intended to capture the existence of the locally conserved patterns. Then the neural response of the protein *p *with respect to a motif *q *is given by:

(3) $$N\left(p,q\right)=\max\left\{s\left(q_{p1},q\right)\ldots \left(q_{pk},q\right)\right\}$$

Now by considering all the *m *motifs in the template layer the information about the protein *p *given the templates can be represented by an *m*-dimensional vector:

(4) $$\text{N}\left(p\right)=\left(\text{N}\left(p,q_{1}\right),\ldots .\text{N}\left(p,q_{m}\right)\right)$$

Our goal is to learn the similarity between the query protein *p_i _*and the proteins in the base dataset such that we can assign the query protein *p_i _*to the GO term(s) associated with the most similar protein *p_j_*. To quantize the similarity between pairs *p_i _*and *p_j_*, we encoded the pair *(p_i_, p_j_) *into a vector N*_(i, j) _*on which we can formulate the *mapping engine *to map the query protein to its most probable GO term. There are two ways to achieve this, by taking the difference between N(*p_i_*) and N(*p_j_*) or by simply concatenating them together. As we found that the former method always gives better performance in our algorithm, we thus let:

(5) $${\text{N}}_{\left(i,j\right)}=\left|\text{N}\left(p_{i}\right)-\text{N}\left(p_{j}\right)\right|$$

$$=\left(\left|N\left(p_{i},q_{1}\right)-N\left(p_{j},q_{1}\right)\right|,\ldots ,\left|N\left(p_{i},q_{m}\right)-N\left(p_{j},q_{m}\right)\right|\right)$$

which is the neural response of the pair *(p_i_, p_j_) *on the templates set *q_1_...q_m_*.

### Derived kernel

We can derive a kernel *K*, which measures the similarity of two protein pairs, from the neural responses. This kernel also gives the similarity of two proteins. Two proteins are similar, if the pair constituted by them is similar to a pair with two similar proteins and vice visa. In the original paper of neural response [28], a linear kernel is defined by inner products of neural responses. Under our setting, the linear kernel for two pairs *(p_i_, p_j_) *and *(p_i_, p_j_) *can be written as

(6) $$K\left(\left(p_{i},p_{j}\right),\left(p_{i^{\prime \prime }},p_{j^{\prime \prime }}\right)\right)=N\left\langle N_{\left(i,j\right)},N_{\left(i^{\prime \prime },j^{\prime \prime }\right)}\right\rangle$$

$$=\sum_{\text{k}=1}^{\text{n}}\text{N}\left(p_{i,}q_{k}\right)\text{N}\left(p_{i^{\prime \prime }},q_{k}\right)+\sum_{\text{k}=1}^{\text{n}}\text{N}\left(p_{j},q_{k}\right)\text{N}\left(p_{j^{\prime \prime }},q_{k}\right)$$

It is well established that the Gaussian kernel usually performs better than the linear kernel for various classification tasks. Thus we had derived a Gaussian kernel with a scale parameter σ, given by

(7) $$K\left(\left(p_{i},p_{j}\right),\left(p_{i^{\prime \prime }},p_{j^{\prime \prime }}\right)\right)=\exp\left\{-\frac{{\left|N_{\left(i,j\right)}-N_{\left(i^{\prime \prime },j^{\prime \prime }\right)}\right|}^{2}}{\sigma^{2}}\right\}$$

### Mapping engine

Finally, a *mapping engine *was built, which defines a function "*f" *lying in the reproducing kernel Hilbert space [42] associated with a positive definite kernel *K *that is derived from the neural responses by inner products (linear kernel) or Gaussian radial basis functions (Gaussian kernel). First, we computed the neural response of all the proteins in the base dataset with respect to the template library in top layer. Similar neural response was computed for the query protein sequence as well. Next we computed the pair wise neural response N*_(i, j) _*between the query sequence *i *and the sequence *j *(*1..n*) in the base dataset. The mapping function *f(N_(i, j)_) *produces a value ranging between 0 to 1 corresponding to similarity between the proteins *p_i _*and *p_j_*. Thus, we can predict the label Y*_(i, j) _*to 1 (similar) if *f(N_(i, j)_) ≥ 0.5*, and Y*_(i, j) _*to 0 (non-similar) if *f(N_(i, j)_) < 0.5 *. Other thresholds besides 0.5 are also allowed. We then assigned the query protein *p_i _*to the GO term/s associated with the protein/s *p_j _*whose label Y*_(i, j) _*was set to 1. In this case the sensitivity of GO term assignments varies with the threshold used (0.5). To overcome this dependency on the threshold, we sorted the proteins in the base dataset into descending order based on their similarity (*f(N_(i, j)_)*) to the query protein. We finally extracted the top 5 GO terms and assign them to the query protein. By doing so, we are not only overcoming the threshold dependency problem but also using the ranking (true value of the *f(N_(i, j)_)*) as the confidence scores for multiple GO terms associated with a single protein.

We used two popular classification engines *viz.*, Support vector Machines (SVM) [43] and Least-Squares classifier [44] as the mapping engine. The main difference between them is, the *loss function *used for training. They use hinge loss and leastsquare loss respectively. The performance of two mapping engines is evaluated in the Results section.

## Competing interests

No relevant disclosures.

## Authors' contributions

HKY collected the data, designed the pipeline, performed evaluations, statistical analysis and wrote the paper. QWX designed and tested the Neural Response algorithm. JW conceptualized the idea, designed the study and wrote the paper. All authors have read and approved the final manuscript.
